# Improving Ovine Behavioral Pain Diagnosis by Implementing Statistical Weightings Based on Logistic Regression and Random Forest Algorithms

**DOI:** 10.3390/ani12212940

**Published:** 2022-10-26

**Authors:** Pedro Henrique Esteves Trindade, João Fernando Serrajordia Rocha de Mello, Nuno Emanuel Oliveira Figueiredo Silva, Stelio Pacca Loureiro Luna

**Affiliations:** 1 Department of Veterinary Surgery and Animal Reproduction, School of Veterinary Medicine and Animal Science, São Paulo State University, Botucatu 05508-270, SP, Brazil; 2 Department of Quantitative Analytics, Escola Superior de Propaganda e Marketing (ESPM), São Paulo 04018-010, SP, Brazil

**Keywords:** animal welfare, artificial intelligence, pain assessment, sheep

## Abstract

**Simple Summary:**

After four decades of studies on methods to assess pain in sheep, a pain scale composed of behavioral items that are fast, robust, and simple to apply was recently developed—the Unesp-Botucatu sheep acute pain scale (USAPS). Scientific evidence suggests that considering the importance of each behavior separately may improve the quality of pain diagnosis; however, this has not yet been studied for animal pain assessment. Therefore, the objective of this study was to investigate whether the implementation of statistical weights using machine learning algorithms improves the discriminatory capacity of the USAPS. A behavioral database, previously collected for USAPS validation, of 48 sheep before and after an abdominal surgical procedure was used. A multilevel binomial logistic regression algorithm and a random forest algorithm were used to establish the statistical weights and classify the sheep as to whether they needed analgesia or not. The quality of the USAPS pain diagnosis weighted by the two algorithms was better than the original version of the instrument. We conclude that considering the importance of each USAPS behavior by the two machine learning algorithms improved the instrument’s ability to differentiate sheep in pain from those free of pain.

**Abstract:**

Recently, the Unesp-Botucatu sheep acute pain scale (USAPS) was created, refined, and psychometrically validated as a tool that offers fast, robust, and simple application. Evidence points to an improvement in pain diagnosis when the importance of the behavioral items of an instrument is statistically weighted; however, this has not yet been investigated in animals. The objective was to investigate whether the implementation of statistical weightings using machine learning algorithms improves the USAPS discriminatory capacity. A behavioral database, previously collected for USAPS validation, of 48 sheep in the perioperative period of laparoscopy was used. A multilevel binomial logistic regression algorithm and a random forest algorithm were used to determine the statistical weights and classify the sheep as to whether they needed analgesia or not. The quality of the classification, estimated by the area under the curve (AUC) and its 95% confidence interval (CI), was compared between the USAPS versions. The USAPS AUCs weighted by multilevel binomial logistic regression (96.59 CI: [95.02–98.15]; *p* = 0.0004) and random forest algorithms (96.28 CI: [94.17–97.85]; *p* = 0.0067) were higher than the original USAPS AUC (94.87 CI: [92.94–96.80]). We conclude that the implementation of statistical weights by the two machine learning algorithms improved the USAPS discriminatory ability.

## 1. Introduction

Concern for the welfare of non-human mammals has increased around the world [1]. Pain is an aversive sensation that directly affects animal welfare, so its accurate diagnosis represents a key point for the promotion of welfare [2]. Despite scientific evidence that mammals are capable of experiencing aversive states as sentient beings, pain is still a welfare issue that demands attention in the life of farm [3] and experimentation animals [4].

On sheep farms, painful procedures are routinely performed, such as castration, tail docking, and mulesing [2,5,6,7]. In addition, sheep are extensively subjected to experimental pain conditions to assess their responses to different noxious stimuli [8,9], including their use as a animal model for osteoarthritis studies due to the similarity of human knee size and anatomy [10,11]. Furthermore, unintentional situations of pain, such as diseases, injuries caused by inadequate housing or handling, mastitis, hoof problems, and dystocia also represent a potential source of pain during the life of sheep [3,12,13]. Therefore, the clear occurrence of pain in sheep requires monitoring to diagnose pain and the need for analgesic treatment, as well as to monitor the duration of the analgesic effect and assess the need for analgesia reintervention [2,7]. However, pain diagnosis is complex due to its multidimensional aspects, given the combination of somatic, cognitive, and emotional components [14] particular to each individual, and thus requires robustly accurate methods for its evaluation.

Since the 1980s, the studies that created ethograms to record the duration and frequency of behavior over a relatively long period during pain situations have reported an increase in abnormal postures and a reduction in decubitus time and normal activities [15,16,17,18]. As behaviors are not pathognomonic to diagnose pain, it is necessary to combine a range of behaviors to fill this demand [7]. However, some behaviors present a very low occurrence for their individual statistical analysis, which demands their grouping into categories, and classification into abnormal behaviors, activity, rest, and others [19,20,21,22,23,24,25,26,27,28,29,30,31,32]. This approach demonstrated that body language is useful to distinguish sheep with pain from those free of pain, but with the disadvantage of giving the same importance to low-occurrence behaviors [7]. Behaviors have different importance depending on the type of procedure [17,28,31]; for example, an increase in the length of time the sheep remains in lateral recumbency occurs after castration using an elastic ring, which does not occur when using other castration methods [26]. Thus, the simple addition of the occurrences of each pain-related behavior in a category, without taking into account its importance for the pain phenomenon, may represent a methodological bias when analyzing different procedures [7] and, consequently, reduce the accuracy of the pain diagnosis.

Knowledge of pain-related behaviors accumulated over the last 40 years has enabled the development of a faster and more practical approach to pain assessment, namely, the Composite Pain Scales or Pain Score Systems. In this method, the various behaviors separated into categories are noted with descriptive items rather than frequency and duration [33,34]. In this sense, an instrument was recently created by our team to perform a quick (4 min) and simple pain assessment in sheep—the Unesp-Botucatu composite scale to assess acute postoperative abdominal pain in sheep (USAPS) [35]. The USAPS underwent a rigorous process of psychometric refinement and validation to analyze the suitability of the instrument’s behavioral items for the purpose, followed by several statistical steps [36,37]. After the process, the USAPS contained only the most robust set of behavioral items for pain assessment and proved to be repeatable (intra-rater reliability) and reproducible by four evaluators (inter-rater reliability), sensitive and specific for pain diagnosis, responsive in perioperative assessments of laparoscopy, with the excellent internal consistency of behavioral items, good accuracy, and discriminatory ability to distinguish sheep suffering pain from pain-free sheep. To our knowledge, to date, the USAPS is the only scale composed of general behaviors, including broad body language, for pain assessment in sheep, that is robustly validated, practical, and simple to apply [35].

The construction of the USAPS was based on behavioral items related to maintenance behaviors that may change when the sheep experiences pain (e.g., activity, locomotion, appetite, interaction) and specific pain or discomfort behaviors (e.g., attention to the affected area, arching the back). In the elaboration of this tool, the same importance was assumed for each of the behaviors, aiding the USAPS to fulfill the role of being an easy instrument without the need for a sophisticated device. On the other hand, the segregation of behaviors into maintenance and specific pain or discomfort signals an intrinsic difference in their classification that requires consideration. Additionally, the behavioral items related to locomotion, activity, and interaction in the USAPS showed greater variation in the perioperative period than those related to appetite, posture, and head position through the descriptive statistics of the analysis of occurrences [35], suggesting different importance for each of the behaviors, regardless of their maintenance, specific pain, or discomfort classification.

A psychometric validation guide discussed the inclusion of weights in the items of an instrument to improve its classifying ability, by adopting theoretical or empirical approaches. Their disadvantages are that the first, by arbitrarily attributing more weight to more important items, is subjective and the second, based on statistical equations, consumes time to perform the calculations [36]. In recent years, technological advances have made computational processes more available, and statistical weightings have been included in human pain assessment instruments [38]. Studies examining patients with various types of pain [39,40,41,42] reported an improvement in the diagnosis when weighting the items in their instruments using a logistic regression algorithm. Weighting the items of a questionnaire for the assessment of neuropathic pain using a canonical analysis algorithm in a multicenter study improved the diagnosis of pain [43]. Otherwise, there was no improvement when applying the random forest algorithm for experimentally induced local skin hypersensitivity in healthy subjects [44]. In veterinary medicine, only one study weighted the items of an instrument to assess colic pain in horses using a theoretical approach [45]; however, empirical weights based on statistical equations have not yet been evaluated in an instrument to assess pain in non-human animals.

Given the above, there is compelling evidence that each behavior used to diagnose pain has different importance. However, the statistical weighting of the behaviors of an instrument to assess pain in sheep has not yet been studied yet. The current study aimed to investigate whether the implementation of statistical weightings using machine learning algorithms improves the discriminatory capacity of the USAPS. We hypothesized that behaviors have different importance for the diagnosis of pain, and therefore, statistical weighting improves the diagnostic ability of the USAPS.

## 2. Materials and Methods

This is an opportunistic study using data from our previous publication [35], approved by the Ethics Committee for the Use of Animals of the School of Veterinary Medicine and Animal Science, São Paulo State University, Botucatu campus (nº 0027/2017), and following the Animal Research standards Reporting of In Vivo Experiments [46]. The experimental procedures presented in the current study were conducted for the creation, refinement, and psychometric validation of the USAPS and are presented in the previous study [35]. The present study includes unpublished analyses from the same database. We understand that database reuse contributes to the four R’s of animal experimentation (reduce, replace, refine, and respect) [47,48] and to the welfare of the sheep.

### 2.1. Dataset

The data were composed of a database with behavioral records of 48 sheep (*Ovis aires*) of three breeds (17 Bergamacia, 18 Lacaune, and 13 Dorper) aged 3.5 ± 1.8 (1.5–6.0) submitted to laparoscopy, with a mean weight of 58.5 ± 17.3 (34–92) kg and diagnosed as healthy by clinical and laboratory tests (hematocrit, plasma protein, glucose, and lactate).

Before starting laparoscopy, 30,000 IU/kg of benzathine penicillin (Pentabiotic^®^, Zoetis, São Paulo, SP, Brazil) was administered intramuscularly (IM). Then, dissociative anesthesia was performed by applying 0.5 mg/kg of diazepam (Compaz^®^, Cristália, Itapira, SP, Brazil) and 5 mg/kg of ketamine (Cetamin^®^, Syntec; Santana de Parnaíba, SP, Brazil) intravenously (IV). For intraoperative analgesia, lumbosacral epidural anesthesia was performed with 0.1 mL/kg of 1% lidocaine without a vasoconstrictor (Xylestesin^®^, Cristália, Itapira, SP, Brazil) and anesthetic infiltration with 2% lidocaine without vasoconstrictor (Xylestesin^®^, Cristália, Itapira, SP, Brazil) at the incision site after the introduction of the trocar. The same experienced surgeon performed all laparoscopies for follicular aspiration and follicular cell replacement using three 5 mm trocars introduced in the retro-umbilical region. Dissociative anesthesia was supplemented during the procedure with 5 mg/kg IV ketamine for those sheep that demonstrated head or limb movement or a 20% increase in heart rate compared to the pre-procedure rate. All sheep received postoperative analgesia 3–4 h after anesthetic recovery with 0.5 mg/kg 2% meloxicam (Maxicam^®^, Ourofino, Cravinhos, SP, Brazil) and 0.2 mg/kg morphine (Dimorf ^®^, Cristália, Itapira, SP, Brazil) IV, separately.

The sheep were filmed at four perioperative time points: before laparoscopy (M1), 3–4 h after recovery from anesthesia and before postoperative analgesia (M2), 1 h after administration of postoperative analgesia (M3), and 24 h after laparoscopy (M4). Four experienced ‘blind’ evaluators randomly rated the four recordings from each sheep. After watching each recording, the evaluators were required to score whether or not they would indicate analgesia for the sheep in the video according to their clinical experience (expert opinion) and then to score the USAPS behavioral items. Tutorial videos of each behavior can be viewed at https://animalpain.org/en/ accessed on 16 August 2022. The expert opinion on whether or not to indicate analgesia was given during the evaluation of the videos after the experiment, so it did not interfere with the clinical conduct practiced during the experiment; all sheep received intraoperative and postoperative analgesia as described above.

In the USAPS, body language related to interaction, activity, locomotion, appetite, head position, and posture was classified by descriptive items composed of three levels. Level ‘0’ indicated normal behaviors (no association with pain), while levels ‘1’ and ‘2’ indicated behaviors associated with pain, with level ‘2’ representing those behaviors related to greater pain severity. The total sum of the USAPS behavioral items was considered to assess pain.

After all the videos had been evaluated once (phase 1), the procedure of watching and evaluating the videos was repeated (phase 2) by the evaluators, after a minimum interval of 30 days. In the previous study, the phases were used to assess intraobserver reliability, but in the current study, they represented a repetition of the behavioral observation.

In summary, the database totaled 1536 observations from 48 sheep, evaluated at four perioperative time points, by four evaluators, and assessed twice. The data are available in the supplementary material of the previous publication [35].

### 2.2. Statistical Description

Statistical analyses were performed by a data scientist (PHET) in R software with the RStudio integrated development environment (Version 4.1.0; 2021-06-29; RStudio, Inc., Boston, MA, USA). The functions and packages used were presented in the format ‘package::function’ corresponding to the computer programming language in R. For all tests, a significance of 5% was considered. All figures were constructed with a color palette distinguishable by colorblind people (ggplot2::scale_colour_viridis_d).

#### 2.2.1. Creation of the Multilevel Binomial Logistic Regression Algorithm

A machine learning algorithm was built with a multilevel binomial logistic regression model (lme4::glmer) using 100% of the sheep in the database and applying the expert opinion as a response variable (no event = no indication of analgesia; event = indication of analgesia). The behavioral items from the USAPS, previously converted into dummy variables (0 = absence and 1 = presence of each level of each item) (fastDummies::dummy_columns), were used as explanatory variables. Each of the 48 sheep has its own characteristics and their individual reactions and behaviors are effects to be controlled. Because we need to classify a general population of sheep, the random effect of sheep was inserted into the model. This means that each sheep had a random effect with a mean of zero and an estimated variance. The same principle was applied to the evaluators and the evaluation time points and phases. Therefore, the 48 sheep, the four evaluators, the four time points, and the two evaluation phases were used as random effects of the model [49].

The full model (containing all fixed and random effects) was compared with the null model (containing only random effects) and with the short model (containing only significant fixed effects and all random effects) by log-likelihood, pseudo-R^2^, and Akaike (AIC) and Bayesian (BIC) information criterion (jtools::summ; stats::logLik; and lmtest::lrtest). To illustrate the importance of each USAPS behavior, the Wald statistic of each explanatory variable of the fixed effects was calculated and presented in a bar plot (ggplot2::ggplot). The Wald statistic is presented by dividing the slope by its standard error. Subsequently, based on the fixed effects coefficients estimated by the algorithm, the probability of each sheep in the database (100% of the sheep) needing analgesia (suffering pain) (stats::predict) was calculated. This probability was used to verify the quality of the pain diagnosis described below.

#### 2.2.2. Creation of the Random Forest Algorithm

A second machine learning algorithm applied was the random forest (caret::trainControl and caret::train) by inserting the expert opinion as the response variable and the dummy variables as explanatory variables, representing the presence or absence of each level of the USAPS behavioral items, as described for the previous algorithm.

Random forest algorithms have a methodological characteristic of identifying patterns sequentially (algorithm training), which may result in a classification rule that is not generalizable to the target population, called overfitting. The random forest algorithm contains hyperparameters that control the complexity of the model since more complex models capture more detail of existing patterns and increase the chances of overfitting. To find the optimal point of complexity of the algorithm, we used a cross-validation technique called grid search, in which several possibilities of parameters are tested in a k-fold structure with a holdout [50].

The holdout used consisted of randomly separating 70% of the sheep from the database used to train the algorithm (training base), and the remaining 30% of the sheep were used to evaluate the quality of the algorithm (test base). Therefore, the random forest was created and trained with the training base using as hyperparameters 5 k-folds, 4 repetitions, 1001 trees, and 2 characteristic variables randomly sampled as candidates for each split in each tree.

The importance of each characteristic variable within the classification of the algorithm was extracted (caret::varImp) and presented in a bar plot (ggplot2::ggplot). Based on the random forest algorithm created with the training base, the probability of each sheep in the training base and in the test base needing analgesia (stats::predict) was calculated and used in the pain diagnosis quality assessment step, as described below.

#### 2.2.3. Quality of Pain Diagnosis

To assess the quality of pain diagnosis, the area under the receiver operating characteristic curve (AUC) and its respective 95% confidence interval were used, obtained with 1001 repetitions per bootstrap (pROC::roc; pROC::ci.auc; and pROC::ci.coords). The AUC represents an index to classify performance, with scores varying from 0 to 100%. Good performance is considered when the AUC > 90% and excellent when the AUC is > 95% [36]. The AUC is attained from the construction of the receiver operating characteristic curve (ROC) based on a predictor variable that represents a reference parameter for the phenomenon and a predictive variable that is the parameter to be tested [50]. All ROC curves were constructed using expert opinion as a predictor variable (no event = no analgesia indication; and event = analgesia indication) and as a predictive variable of the sum of the USAPS (original USAPS) or the probability of the sheep needing analgesia based on each of the algorithms. For the multilevel binomial logistic regression algorithm, the ROC curves were constructed with 100% of the sheep and with the test base, while for the random forest algorithm, the ROC curves were constructed from the training and test base. To obtain the AUC, it is necessary to establish a cut-off point for each predictive variable based on the Youden index (YI), comprising the sum of the specificity and sensitivity minus 1, calculated for each value of the predictive variable. This index indicates the concomitant maximum specificity and sensitivity, attributing similar importance of specificity and sensitivity to the cut-off point [36]. The ROC curve and YI were constructed exclusively for the calculation of the AUC.

The AUCs of the original and weighted USAPS from each algorithm were compared across all databases run with each algorithm by the DeLong test (pROC::roc.test). Furthermore, the AUC of the short model and full model of the multilevel binomial logistic regression algorithm with 100% of the sheep, as well as the AUC of both algorithms using the test base were also compared with the same test.

Finally, to illustrate the relationship between the expert opinion and multilevel binomial and random forest logistic regression algorithms, a multiple correspondence analysis was carried out (MCA; FactoMineR::MCA) with the dummy variables using 100% of the sheep and another one with the training base, respectively. For each MCA, a two-dimensional perceptual map was constructed, in which the shape of the point indicating each observation represented the expert opinion and the color palette represented the probability of the sheep needing analgesia according to each algorithm (ggplot2::ggplot). The same perceptual map was also built interactively and with three dimensions to maximize the reader’s experience in viewing the results (plotly::plot_ly). For a qualitative evaluation, three perceptual maps were built, applying different colors in the observations to highlight the distribution of time points, evaluators, and phases, and a final perceptual map was built with the distribution of the dummy variables of the behavioral items of the USAPS (factoextra::fviz_mca and ggpubr::ggarrange).

## 3. Results

### 3.1. Creation of the Multilevel Binomial Logistic Regression Algorithm

Compared to the null model (containing only random effects), the twice-smaller AIC and BIC parameters and significantly smaller log-likelihood found in the full model (containing all fixed and random effects) indicate a better adjustment (Table 1). The pseudo-R^2^ > 0.75 of the full model indicates that two-thirds of the data variation was explained by the proposed model. There was low variation in random effects, indicating adequate data consistency. These results suggest an adequate fit of the multilevel binomial logistic regression algorithm.

All slope coefficients were positive (estimate), indicating that the display of these behaviors is related to the indication of analgesia (pain), and demonstrating coherence with the structure of the pain assessment instrument used (USAPS). Most slope coefficients were significant (*p* < 0.05). However, ‘Lying down with head resting on the ground or close to the ground’ showed only a significant trend (*p* = 0.0837), and ‘Head position 1‘ and ‘2’, ‘Appetite 2’, and ‘Arched back’ showed *p* > 0.10. The short model (excluding non-significant angular coefficients) showed a slight decrease in the AIC, BIC, and pseudo-R^2^ of the fixed effects, the same value of total pseudo-R^2^, and as non-significant for the difference in log-likelihood in relation to the full model, demonstrating that there was no substantial improvement in the fit of the model when the non-significant slope coefficients of the full model were disregarded.

The probability (P) of a sheep requiring analgesia according to the multilevel binomial logistic regression algorithm can be calculated by a linear equation using the fixed effects of the model. First, it is necessary to calculate the logit represented by the Greek letter η and calculated with a linear combination of the coefficients and variables of the logistic regression, as shown in Equation (1), where α represents the linear coefficient, β indicates the slope coefficient, and X represents each of the dummy variables (behaviors).

(1) $$\eta =\;\alpha +\beta_{1}{}_{X1}+{\;\beta }_{2}{}_{X2}+{\;\beta }_{3}{}_{X3}+{\;\beta }_{4}{}_{X4}+{\;\beta }_{5}{}_{X5}+{\;\beta }_{6}{}_{X6}+{\;\beta }_{7}{}_{X7}+{\;\beta }_{8}{}_{X8}+{\;\beta }_{9}{}_{X9}+{\;\beta }_{10}{}_{X10}+{\;\beta }_{11}{}_{X11}+{\;\beta }_{12}{}_{X12}+{\;\beta }_{13}{}_{X13}+{\;\beta }_{14}{}_{X14}$$

Thus, as the α and β of all variables have already been estimated (Table 1), it remains only to replace each dummy variable (behavior, as ‘Interaction 1′ for example) in Equation (2), while ‘1’ indicates when the behavior is observed or ‘0’ when absent.

(2) $$\begin{array}{c} \eta =-4.0592+1.4327\;\times \;\mathrm{Interaction}\;1+2.6128\;\times \;\mathrm{Interaction}\;2\;+\\ 2.2143\;\times \;\mathrm{Locomotion}\;1+2.8235\;\times \;\mathrm{Locomotion}\;2\;+\\ 0.1648\;\times \;\mathrm{Head}\;\mathrm{Position}\;1+0.1400\;\times \;\mathrm{Head}\;\mathrm{Position}\;2\;+\\ 1.5283\;\times \;\mathrm{Appetite}\;1+0.4517\;\times \;\mathrm{Appetite}\;2\;+\\ 1.9310\;\times \;\mathrm{Activity}\;1+1.6895\;\times \;\mathrm{Activity}\;2\;+\\ 0.5527\;\times \;\mathrm{Arched}\;\mathrm{Back}\;+0.6284\;\times \;\mathrm{Extends}\;\mathrm{Head}\;+\\ 0.7387\;\times \;\mathrm{Lying}\;\mathrm{Down}+2.0622\;\times \;\mathrm{Moves}\;\mathrm{Tail} \end{array}$$

Finally, it is necessary to include the logit (η) in Equation (3) to obtain the probability (P) of the sheep needing analgesia. The e represents the Euler number ≅ 2.718281828459045235360287.

(3) $$P_{\left(\mathrm{need}\;\mathrm{analgesia}\right)}=\frac{1}{1+e^{-\eta }}$$

**Figure 1:**
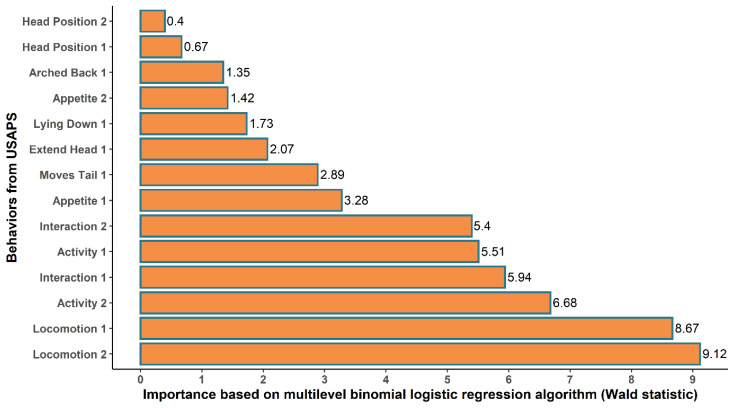
Importance of each behavior from USAPS based on the Wald statistic of the fixed effects in the multilevel binomial logistic regression algorithm using the expert opinion (no-event = no indication of analgesia; event = indication of analgesia) as the predictor variable.

The Wald statistic highlighted ‘Activity 2’, ‘Locomotion 1 and 2’, and ‘Interaction 1 and 2’ as the five most important behaviors for the multilevel binomial logistic regression algorithm, suggesting that the contribution of each behavior has different importance as to the need for analgesia (Figure 1).

### 3.2. Creation of the Random Forest Algorithm

The probability of a sheep needing analgesia according to the random forest algorithm is not easily expressed in an equation due to its complexity and non-linearity.

**Figure 2:**
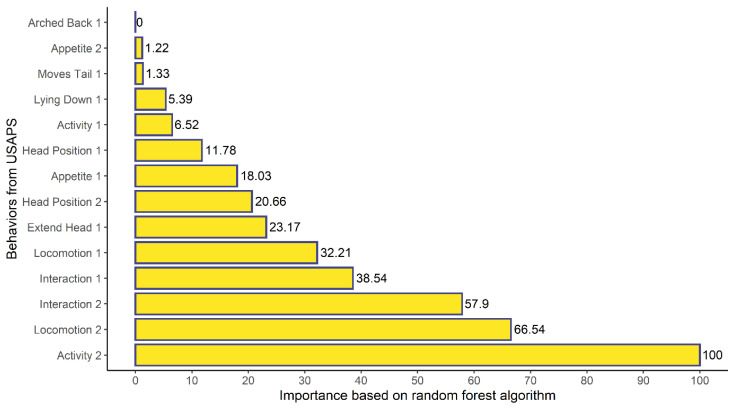
Importance of each behavior from USAPS based on the random forest algorithm using the expert opinion (no event = no indication of analgesia; event = indication of analgesia) as the predictor variable.

‘Activity 1 and 2’, ‘Locomotion 1 and 2’, and ‘Interaction 1’ were the five most important behaviors for the random forest algorithm, indicating that the contribution of each behavior is heterogeneous in the classification of needing or not analgesia in line with the expert opinion (Figure 2).

### 3.3. Quality of Pain Diagnosis

For the multilevel binomial logistic regression algorithm, the specificity and sensitivity were numerically superior, and the AUC was significantly higher in the weighted USAPS compared to the original one (Table 2). The AUC calculated for the short model (96.77 [95.93–97.60]%) was statistically equivalent (*p* = 0.5415) to that of the full model (96.83 [95.98–97.68]%). These findings provide evidence of the diagnostic improvement when the importance of each behavior is considered, including those behaviors that showed non-significant angular coefficients.

For the random forest algorithm, the specificity and sensitivity were numerically superior, and the AUC was significantly higher in the USAPS weighted by the random forest algorithm in relation to the sum of the original USAPS in the training base and in the test base (Table 3). These findings demonstrate the improvement in diagnostic capacity when the importance of each USAPS behavior is considered.

Comparing the algorithms using the test base, the AUC calculated by the multilevel binomial logistic regression (96.59 [95.02–98.15]%) was statistically equivalent (*p* = 0.5684) to that estimated by the random forest algorithm (96.28 [94.17–97.85]%). The ranking of importance of behavioral items was mostly similar between the algorithms, except for ‘Head Position 2’ and ‘1’, ‘Moves Tail’, and ‘Activity 1’ which showed a difference greater than ±3 positions in the ranking (Table 4).

**Figure 3:**
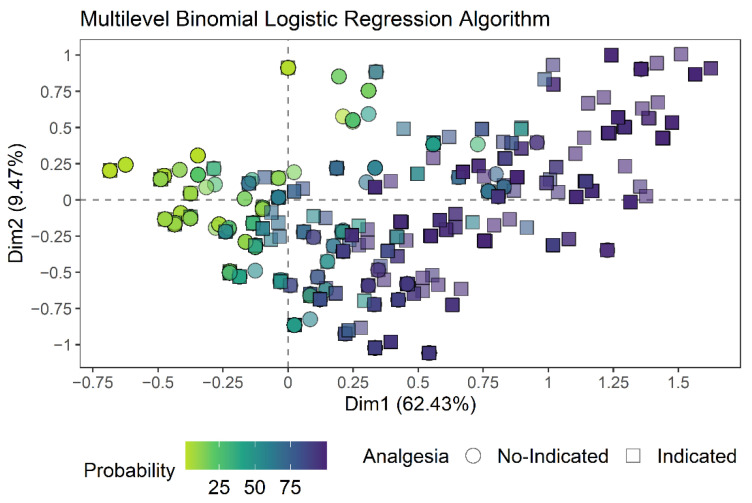
Two-dimensional perceptual map of the multiple correspondence analysis showing the dispersion of expert opinion of no-indication or indication to apply analgesia and probability of sheep needing analgesia according to the multilevel binomial logistic regression model (Circles and squares indicate each evaluation; the greater the number of dark squares and the greater the number of light circles, the greater the accuracy of the algorithm).

**Figure 4:**
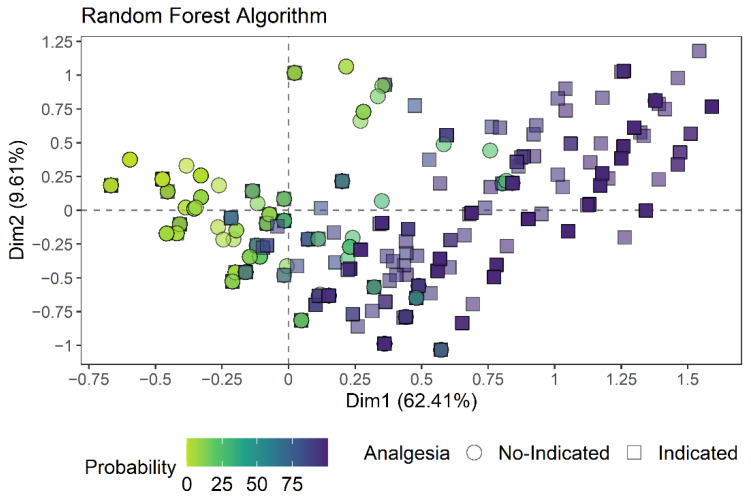
Two-dimensional perceptual map of the multiple correspondence analysis showing the dispersion of expert opinion of no-indication or indication to apply analgesia and probability of sheep needing analgesia according to the random forest algorithm (Circles and squares indicate each evaluation; the greater the number of dark squares and the greater the number of light circles, the greater the accuracy of the algorithm).

**Figure 5:**
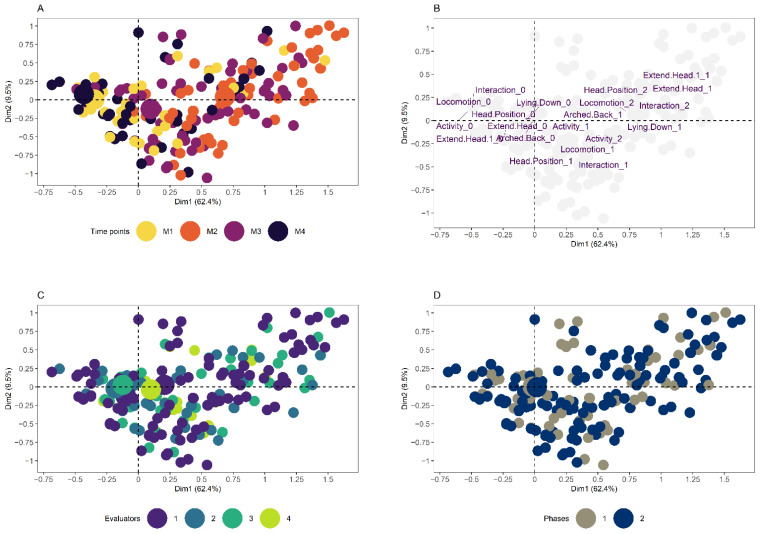
Two-dimensional perceptual map of the multiple correspondence analysis showing the dispersion of the time points (**A**), behavioral items of the USAPS (**B**), evaluators (**C**), and phases (**D**) based on 100% of the sheep dataset [Time points: before laparoscopy (M1), 3–4h after anesthetic recovery, and before postoperative analgesia (M2), 1 h after the administration of postoperative analgesia (M3) and 24 h after laparoscopy (M4); Smaller circles indicate each evaluation and larger circles indicate the centroid)].

The majority of evaluations in which analgesia was indicated by the expert opinion (square symbol) are colored by darker colors (greater probability of needing analgesia by the algorithms), while the majority of evaluations not indicated as needing analgesia (circle symbol) are colored by lighter colors (less likely to need analgesia by the algorithms) (Figure 3 and Figure 4), that is, the greater the number of dark squares and the greater the number of light circles, the greater the accuracy of the algorithm. In addition, the distribution of evaluations in which the sheep received more indications of analgesia according to the expert opinion and a greater probability of needing analgesia according to the algorithms was coincident with the centroid of the expected time point of greatest pain (M2; large orange circle), visible in the upper right quadrant (Figure 5A). The centroid represents the center of gravity of a polygon (flat figure) that can be found in our perceptual map when a line is drawn from the points of the same color, forming the polygon. In the interactive figure, it is possible to observe the separation of the data into two clouds of points when the figure is manually rotated (Figures S1 and S2), in which the smallest cloud corresponds to the observations of sheep at the expected time point of greatest pain (M2).

The expert opinion and the probability of needing analgesia by the algorithms were also located coincidentally with the centroid of the second expected time point of greatest pain (M3; large purple circle), observable in the lower right quadrant (Figure 5A). The distribution of the USAPS behavioral items at levels ‘1’ and ‘2’ (behaviors associated with pain) was mostly located in the upper and lower right quadrants, where there was a greater indication of analgesia by the expert opinion and a greater probability of needing analgesia by the algorithms (Figure 5B). Finally, the distribution of observations taking into account the evaluators (Figure 5C) showed a small difference between the centroids located close to the origin (convergence of the y and x-axis of the graph), and the phases (Figure 5D) because their centroids were almost completely overlapped and located at the origin on the map. These results illustrate the excellent diagnostic capability of the algorithms.

## 4. Discussion

The pioneering efforts of this study lie in developing and implementing statistical weightings in the behavioral items of an instrument for pain assessment in animals, specifically in sheep (USAPS). The two machine learning algorithms applied showed that USAPS behaviors have distinct levels of importance for diagnosing sheep that do or do not need analgesia, according to the expert opinion. Furthermore, the diagnostic capacity of the USAPS applying the two algorithms was higher compared to the original USAPS. These results confirm our initial hypothesis that the importance of the behaviors used to diagnose pain is different and that the consideration of the statistical weighting of this importance improves the USAPS’ discriminatory ability. The advances achieved with the weighted USAPS have the potential to improve the monitoring of pain in sheep on farms and in experiments that induce pain or that compare the efficiency of different analgesic treatments.

In creating the multilevel binomial logistic regression algorithm, some behaviors of the full model (all USAPS behaviors) showed non-significant slope coefficients (‘Lying down with head resting on the ground or close to the ground’, ‘Head position 1’ and ‘2’, ‘Appetite 2’, and ‘Arched back’). Despite this, when comparing the short model (excluding these five behaviors) with the full model, the adjustment parameters of the modeling were similar, and the AUCs of both models were statistically equivalent, suggesting a similar diagnostic capacity between the models. This can be partially explained by the lower frequency of occurrence and/or lower relevance of these behaviors. Other studies have reported changes in appetite [22,23,27], longer times of the sheep remaining lying down [22,23,24,27], with the head down [24,27], and arching the back [22,23,24,27] associated with painful situations in sheep. For these reasons, and also because all USAPS behaviors have been robustly refined and psychometrically validated [35], we chose to use the full model with all USAPS behaviors, which demonstrated good performance and excellent diagnostic ability (AUC > 95%).

The random forest algorithm performed well on the training base with an excellent discriminatory ability (AUC > 95%), demonstrating that the algorithm was created and trained properly with the selected hyperparameters. On the test base, the algorithm also showed good performance, with only a slight decrease in the AUC compared to that estimated using the training base. This was expected because they are different databases [50] and demonstrates the good performance of the random forest algorithm for pain diagnosis.

Regarding the difference in the importance of behaviors for the diagnosis of pain, the five most important behaviors according to the multilevel binomial logistic regression algorithm (‘Activity 2’, ‘Locomotion 1 and 2’, ‘Interaction 1 and 2’) and the random forest algorithm (‘Activity 1 and 2’, ‘Locomotion 1 and 2’, ‘Interaction 1’) were similar. In our validation study, the behavioral items corresponding to activity, locomotion, and interaction showed the highest loading values (λ) in the principal component analysis, demonstrating greater variation and suggesting the greater importance of these behaviors [35]. These similarities suggest that if weights were calculated from the λ of principal component analysis, as conducted in pain scores in humans with pancreatic cancer [51], the results of the importance attributed to each behavior would be similar, but with the limitation of weighting behavioral items grouped into categories of various behaviors instead of individually weighting each behavior as performed in the current study. Furthermore, in our previous study, the internal consistency calculated by Cronbach’s alpha and McDonald’s omega coefficients was reduced when these items were excluded, evidencing the relevance of items related to activity, locomotion, and interaction to the instrument as a whole [35,35]. These results demonstrate that the findings of the present study corroborate the psychometric properties of the previously validated USAPS [35,35].

Interestingly, the most important behaviors for the diagnosis of pain for both algorithms were those of maintenance related to activity, locomotion, and interaction, while the specific behaviors of pain or discomfort were of lesser importance. In our previous study, specific pain behaviors had a lower occurrence and almost exclusively only at the time point of greatest pain, while changes in maintenance behaviors were more frequent at all perioperative time points [35]. Sheep submitted to mulesing without the administration of non-steroidal anti-inflammatory drugs (NSAID) walked less (*p* < 0.01) and remained more stooped (*p* < 0.05) in relation to those that received NSAID; however, there was a greater statistical difference in behavior maintenance related to locomotion than in the specific pain behavior of arching the back [18]. Furthermore, abnormal behaviors also showed low occurrence in other studies [7,16,17,18]. In this way, less intense pain modifies maintenance behaviors, and as the pain intensifies, the display of abnormal behaviors, specific to pain or discomfort occurs. This reinforces the relevance of statistically weighting the behaviors used to recognize pain in sheep.

The statistically superior USAPS AUC weighted by the two machine learning algorithms compared to the original USAPS confirms our hypothesis that the USAPS’ discriminatory ability improves when the importance of each behavior was considered. These results demonstrate an improvement in the quality of the diagnosis from good (AUC > 90%) to excellent (AUC > 95%) with the implementation of the weighting [36]. Studies have also reported an improvement in the accuracy of instruments for pain assessment in human medicine after weighting the items with a logistic regression algorithm [39,40]. A numerical increase in the AUC from 76 to 77% was reported after weighting using canonical discriminant analysis of items in a questionnaire designed to assess neuropathic pain in human patients [43]. On the other hand, the pioneering study on the inclusion of statistical weighting by logistic regression in an instrument to assess pediatric pain did not observe an improvement in the AUC of the weighted instrument compared to the original version [41]. When the weights of the items are similar (same importance) or when the instrument has many items, it is expected that the weighting does not provide improvements, this fact suggests that the instrument should be first refined before being weighted [36]. In this sense, after three years, the same authors reported that, after excluding redundant items, the AUC improved from 95 to 97% after weighting with logistic regression [42]. Subsequently, several other studies applying the weighted instrument confirmed its efficiency [52,53,54,55].

In the present study, most behaviors showed similar importance in both algorithms, with a variation of ±3 positions in the importance ranking. Among the behaviors that changed more than ±3 positions in the ranking, the majority changed their position in the final half of the ranking (from the 7th to the 14th position), except for ‘Activity 1’, which occupied the 10th position in the algorithm’s importance ranking of multilevel binomial logistic regression and the 5th position for the random forest algorithm. This can be partially explained by the fact that binomial logistic regression diagnoses pain based on a linear coefficient and slope coefficients using all predictive variables (behaviors) together, following a linear pattern and simple interpretation. Otherwise, random forest is based on the Gini index of several decision trees, randomly using some of the predictive variables in each tree and following a non-linear and complex pattern known as a ‘black box’ [50]. It should also be considered that the multilevel binomial logistic regression algorithm was built with 100% of the sheep in the database, while the random forest algorithm was built with 70% of the sheep due to the need for cross-validation. Furthermore, particularly in our study, the multilevel binomial logistic regression algorithm considered the repetitions of the 48 sheep, four moments, four evaluators, and two phases as random effects of the multilevel modeling, while the random forest algorithm considered all the data together without this differentiation in levels. Therefore, it is natural that the algorithms assume different importance for the same behavior.

As both algorithms showed a statistically equivalent AUC, by the law of parsimony, we suggest that the multiple binomial logistic regression algorithm may be more useful and reproducible in our case due to its simplicity. We tested two algorithms that we considered suitable for the architecture of the data of our study; however, this represents only the first step towards the statistical weighting of instruments for pain assessment in animals. In the future, other methodologies to statistically weight behaviors should be analyzed, such as canonical discriminant analysis, artificial networks, support vector machines, and a Bayesian classifier, already applied in human medicine [38].

One of the main challenges in pain assessment in non-human animals is the lack of a gold standard methodology to objectively recognize pain since these animals do not verbally communicate the level of their pain in a way that we clearly understand [7]. We determined whether or not the sheep required analgesia based on the opinion of an expert, which can be understood as a limitation of the study due to the subjectivity of the method. In our case, another option would be to use the time points before (M1) and immediately after recovery from anesthesia from laparoscopy (M2) to determine pain-free sheep (pre-laparoscopy) from those with pain (post-laparoscopy). However, in our previous study, some sheep showed USAPS scores below the optimal cut-off point, indicative of analgesia even at the expected time point of greatest pain (M2), in addition to which, some sheep presented a USAPS score above the cut-off 24 h after laparoscopy (M4). Furthermore, other studies have shown that pain-associated behaviors may persist longer than 24 or 48 h after the painful stimulus [7,23,25]. These variations can be partially explained by the individuality of the painful sensation and the trans- and postoperative analgesic efficacy. Therefore, in order not to neglect these particularities, we chose to use expert opinion throughout the four perioperative time points. To the best of our knowledge, the expert opinion currently appears to provide the best possible results for determining animals with pain from pain-free animals [56,57,58,59,60,61,62,63,64,65,66,67,68], but we recognize that this is still a challenge that requires attention.

The calculation of the probability of needing analgesia provided by the algorithms of our study may represent a limitation for the application of statistical weights [36] in cases where the calculation is not performed automatically using an application or website. In our view, the cost of calculating the probability is offset by the benefit of diagnostic accuracy, so future steps should focus on automating the calculation, which is already underway by our team (www.animalpain.org, accessed on 14 August 2022). Another limitation of this study is that adult sheep were evaluated exclusively after laparoscopy, and there is evidence that pain-associated behaviors may be procedure-specific [7,17,26,28,31]. Furthermore, the age of the animal could be another confounding factor, as young sheep decrease their jumping behavior, like rabbits, when they experience pain [20,22,27,29], while this behavior is rare in adult sheep [35]. Another confounding factor is that the time of day influences the maintenance behaviors used in pain assessment in horses; horses walk and look out of the stall window more and rest less during the day than at night when stabled in a veterinary hospital [69]. Although some factors influence pain-related behaviors, the increase in the number of parameters makes the algorithm more complex and less generalizable, so the cost–benefit balance of including these effects or any others (e.g., age, weight, breed, time of day, breeding destination) needs to be studied carefully in the future. We understand that the approach carried out in this study met its role of demonstrating the importance of statistical weights and that the algorithm created can be continuously improved in the future through deep learning. To paraphrase the renowned statistician George Edward Pelham Box, “essentially, all models are wrong, but some are useful” [70]. Models try to summarize and simplify reality and intrinsically miss some details [71], especially when dealing with models applied to explain complex phenomena such as pain [7].

In practice, our findings demonstrate that the inclusion of weights contributes to a better diagnosis of sheep suffering or not pain. Furthermore, the understanding of a percentage from 0 to 100 provided by the USAPS weighted version is easier to interpret by the lay public than a sum of 0 to 12 as proposed in the original USAPS version.

We understand that the USAPS, after being refined, validated, and weighted, is a more robust instrument to assess pain in sheep than the original version and, in the future, artificial intelligence could be developed for automatic recognition of the instrument’s behaviors, as performed with instruments that exclusively analyze facial expressions in sheep [72,73]. It is noteworthy that for automatic recognition to achieve its best diagnostic performance, the instrument needs to be in its best version, and, in this sense, statistical weighting plays a fundamental role in achieving the best possible instrument.

Finally, weighting could be implemented in the future in recently validated instruments for pain assessment that use full body language and/or the face in other non-human mammals such as felines [56,57,61,62,74], bovines [63,64], swine [65,75], horses [66,67], donkeys [58,68], and lagomorphs [59,60].

## 5. Conclusions

We conclude that the implementation of weighting based on the two machine learning algorithms applied demonstrated distinct levels of the importance of the USAPS pain-associated behaviors to classify sheep as needing or not needing analgesia according to expert opinion. The diagnostic capacity of the weighted USAPS applying the two algorithms was improved compared to the original USAPS. Our results support our initial hypothesis that the importance of the behaviors used to diagnose pain is heterogeneous and that the consideration of weighting improves the discriminatory ability of the USAPS.

## Figures and Tables

**Table 1:** Findings based on a multilevel binomial logistic regression algorithm using the expert opinion (no-event = no indication of analgesia; event = indication of analgesia) as the predictor variable.

N = 1536	Slope Coefficient (β)			
Fixed Effects	Estimate	SE	Z-Value	*p*-Value
Linear coefficient (α)	−4.0592	0.3949	−10.2799	8.69^−25^ ****
Interaction score				
(0) Active, attentive to the environment, interacts and/or follows other animals				
(1) Apathetic: may remain close to other animals, but interacts little	1.4327	0.2411	5.9415	2.83^−9^ ****
(2) Very apathetic: isolated or not interacting with other animals, not interested in the environment	2.6128	0.4838	5.4004	6.65^−8^ ****
Locomotion score				
(0) Moves about freely, without altered locomotion; when stopped, the pelvic limbs are parallel to the thoracic limbs				
(1) Moves about with restriction and/or short steps and/or pauses and/or lameness; when stopped, the thoracic or pelvic limbs may be more open and further back than normal	2.2143	0.2555	8.6650	4.51^−18^ ****
(2) Difficulty and/or reluctant to stand up and/or not moving and/or walking abnormally and/or limping; may lean against a surface	2.8235	0.3096	9.1207	7.46^−20^ ****
Head position score				
(0) Head above the withers or eating				
(1) Head at the height of withers	0.1648	0.2469	0.6677	0.5043
(2) Head below the withers (except when eating)	0.1400	0.3513	0.3984	0.6903
Appetite score				
(0) Normorexia and/or rumination present				
(1) Hyporexia	1.5283	0.4657	3.2821	0.0010 ***
(2) Anorexia	0.4517	0.3172	1.4239	0.1545
Activity score				
(0) Moves normally				
(1) Restless, moves more than normal, or lies down and stands up frequently	1.9310	0.3507	5.5059	3.67^−8^ ****
(2) Moves less frequently or only when stimulated using a stick or does not move	1.6895	0.2530	6.6781	2.42^−11^ ****
Posture score				
Arched back	0.5527	0.4081	1.3543	0.1756
Extends the head and neck	0.6284	0.3031	2.0729	0.0382 **
Lying down with head resting on the ground or close to the ground	0.7387	0.4271	1.7295	0.0837 *
Moves the tail quickly (except when breastfeeding) and repeatedly and/or keeps the tail straight (except to defecate/urinate)	2.0622	0.7126	2.8940	0.0038 ***
Random effects	Variance	SD	Groups	
Sheep (intercept)	8.6422^−9^	9.2963^−5^	48	
Observers (intercept)	5.3755^−2^	2.3185^−1^	4	
Moments (intercept)	3.0738^−1^	5.5442^−1^	4	
Phases (intercept)	9.1439^−8^	3.0239^−4^	2	
Model parameters	Full model	Null model	Short model	
Log-Likelihood (df)	−325.48 (19)	−683.97 (5)	−329.22 (14)	
P-value Log-Likelihood vs. Full Model	-	<2.2^−16^ ****	0.1874	
AIC	688.96	1377.96	686.44	
BIC	790.36	1404.64	761.15	
Pseudo-R² (fixed effects)	0.76	-	0.75	
Pseudo-R² (total)	0.78	0.60	0.78	

AIC is Akaike information criterion; BIC is the Bayesian information criterion; SE is standard error; SD is standard deviation; df is degrees of freedom; **** is *p* < 0.001; *** is *p* < 0.01; ** is *p* < 0.05; * is *p* < 0.10; Full model includes all fixed and random effects; Null model includes only random effects; Short model includes only significant fixed effects and all random effects.

**Table 2:** Optimal cut-off, specificity, sensitivity, and area under the curve from receiver operating characteristic curve of the original USAPS and weighted USAPS from multilevel binomial logistic regression algorithm (no event = no indication of analgesia; event = indication of analgesia).

Dataset	Parameters	Original USAPS (Total Sum)	Weighted USAPS (Probability to Need Analgesia Based on Logistic Regression)	*p*-Value
100%	Optimal cut-off	03.50 (03.50–04.50)	43.88 (29.61–61.38)	-
	Specificity	87.67 (85.50–93.49)	91.21 (87.21–94.43)	-
	Sensitivity	91.97 (85.61–93.94)	92.88 (87.88–96.06)	-
	AUC	95.32 (94.30–96.35)	96.83 (95.98–97.68)	1.381^−9^
Test data (30%)	Optimal cut-off	03.50 (03.50–04.50)	59.63 (43.14–66.67)	-
	Specificity	86.07 (80.74–93.03)	92.21 (86.89–95.90)	-
	Sensitivity	92.16 (83.82–96.08)	92.16 (87.25–96.57)	-
	AUC	94.87 (92.94–96.80)	96.59 (95.02–98.15)	4.891^−4^

AUC is area under the curve; comparison between two AUCs was conducted with the DeLong test; the optimal cut-off point was determined by the Youden index (Specificity + Sensitivity—1).

**Table 3:** Optimal cut-off, specificity, sensitivity, and area under the curve from the receiver operating characteristic curve of the original USAPS and weighted USAPS from random forest algorithm (no event = no indication of analgesia; event = indication of analgesia).

Dataset	Parameters	Original USAPS (Total Sum)	Weighted USAPS (Probability to Need Analgesia Based on Random Forest)	*p*-Value
Training data (70%)	Optimal cut-off	03.50 (03.50–04.50)	42.21 (20.68–64.09)	-
	Specificity	88.92 (86.55–94.94)	94.62 (90.82–96.99)	-
	Sensitivity	91.45 (85.09–94.08)	93.42 (90.13–96.49)	-
	AUC	95.47 (94.25–96.69)	97.50 (96.56–98.45)	1.822^−9^
Test data (30%)	Optimal cut-off	03.50 (03.50–04.50)	35.41 (35.26–65.13)	-
	Specificity	86.07 (80.74–93.03)	89.34 (85.25–93.85)	-
	Sensitivity	92.16 (83.82–96.08)	95.10 (90.69–98.04)	-
	AUC	94.87 (92.94–96.80)	96.28 (94.17–97.85)	0.0067

AUC is area under the curve; comparison between two AUCs was conducted with the DeLong test; the optimal cut-off point was determined by the Youden index (Specificity + Sensitivity—1).

**Table 4:** USAPS behavior importance ranking based on two machine learning algorithms.

Behavioral Items	Ranking		
	Multiple Binomial Logistic Regression	Random Forest	Delta
‘Activity 2′	1st	3rd	2
‘Locomotion 2′	2nd	1st	−1
‘Interaction 2′	3rd	6th	3
‘Interaction 1′	4th	4th	0
‘Locomotion 1′	5th	2nd	−3
‘Extend Head’	6th	9th	3
‘Head Position 2′	7th	14th	7
‘Appetite 1′	8th	7th	−1
‘Head Position 1′	9th	13th	4
‘Activity 1′	10th	5th	−5
‘Lying Down’	11th	10th	−1
‘Moves Tail’	12th	8th	−4
‘Appetite 2′	13th	11th	−2
‘Arched Back’	14th	12th	−2

The delta represents the difference in ranking position between algorithms.

## Data Availability

The data presented in this study are available in the supplementary material according to “MDPI Research Data Policies” at https://www.mdpi.com/ethics.

## Supplementary Materials

The following supporting information can be downloaded at: https://www.mdpi.com/article/10.3390/ani12212940/s1, Figure S1: Interactive three-dimensional perceptual map of the multiple correspondence analysis showing the dispersion of expert opinion of no-indication or indication to administer analgesia and the probability of sheep needing analgesia according to the multilevel binomial logistic regression algorithm (Circles and squares indicate each evaluation; the greater the number of dark squares and the greater the number of light circles, the greater the accuracy of the algorithm); Figure S2: Interactive three-dimensional perceptual map of the multiple correspondence analysis showing the dispersion of expert opinion of no-indication or indication to administer analgesia and the probability of sheep needing analgesia according to the random forest algorithm (Circles and squares indicate each evaluation; the greater the number of dark squares and the greater the number of light circles, the greater the accuracy of the algorithm).
